# Antibiotic Cement in Arthroplasty: A Meta-analysis of Randomized Controlled Trials

**DOI:** 10.7759/cureus.7893

**Published:** 2020-04-29

**Authors:** Seper Ekhtiari, Thomas Wood, Raman Mundi, Daniel Axelrod, Vickas Khanna, Anthony Adili, Mitchell Winemaker, Mohit Bhandari

**Affiliations:** 1 Orthopaedic Surgery, McMaster University, Hamilton, CAN; 2 Surgery, McMaster University, Hamilton, CAN; 3 Orthopaedics, McMaster University, Hamilton, CAN

**Keywords:** arthroplasty, infection, cement, periprosthetic joint infection, total joint arthroplasty

## Abstract

Introduction

Periprosthetic joint infection (PJI) following arthroplasty surgery is a devastating complication. Antibiotic cement has been proposed as a way to reduce PJI rates. The aim of this systematic review and meta-analysis was to review all of the available randomized controlled trial (RCT) evidence on the use of antibiotic cement in arthroplasty.

Methods

PubMed, MEDLINE, and Embase were searched. All records were screened in triplicate. Eligible RCTs were included. Data regarding study characteristics, patient demographics, and rates of superficial and deep infection were collected. The risk of bias was assessed using the Cochrane Risk of Bias Assessment Tool 2.0.

Results

Five RCTs were included (n = 4,397). Four studies compared antibiotic cement to plain cement while one study compared high-dose dual-antibiotic (HDDA) cement to low-dose single-antibiotic (LDSA) cement. The mean age of included patients was 76.4 years (range: 68-83). There was no significant difference in superficial infection rates between antibiotic and plain cement (odds ratio (OR): 1.33, 95% Confidence Interval (CI): 0.77-2.30, p = 0.3). There was a large but non-significant reduction in deep infection rates for antibiotic cement (OR: 0.20, 95%CI: 0.03-1.32, p = 0.09). There was a significantly lower rate of infection with HDDA as compared to LDSA (OR: 0.31, 95% CI: 0.09-0.88, p = 0.041).

Conclusion

The available evidence from RCTs reveals a potential benefit for antibiotic cement in arthroplasty surgery, though this difference is non-significant and highly imprecise. Furthermore, HDDA cement was significantly more effective than LDSA cement. There is a need for large, pragmatic trials on this topic.

## Introduction

Across the United States and Canada alone, over one million primary total hip arthroplasty (THA) and total knee arthroplasty (TKA) procedures are performed on an annual basis and the incidence of these procedures is steadily increasing [1-3]. The success of joint replacement surgery in restoring patient function and improving quality of life has been well accepted [4-5]. In fact, THA has been previously deemed the “orthopedic operation of the century” [6].

Complications after hip and knee arthroplasty significantly undermine the success of these elective procedures. Periprosthetic joint infections (PJIs), in particular, remain one of the most devastating complications following hip and knee replacement for physicians and patients alike, as these infections result in increased patient morbidity and mortality, psychological hardship, and a significantly increased financial burden to health care systems [7-9]. Given the heightened concern for PJIs shared universally by surgeons, a variety of peri- and intraoperative practice patterns have emerged to mitigate infection risk. The evidence to inform many of these practices, however, remains equivocal and controversial [10].

The use of antibiotic cement has gained particular attention, despite conflicting evidence for its efficacy in preventing PJIs [11-13]. It remains crucial to clarify the efficacy of bone cement, however, as unnecessary use of antibiotic-laden bone cement (ALBC) leads to needless health care costs and potential concerns with antimicrobial stewardship, though limited data exists on this latter issue specifically. Conversely, if ALBC is truly effective in decreasing infection risk, even modest reductions in infection rates can have a significant impact given the number of joint replacements performed annually.

As such, the objective of the current study was to perform a systematic review and meta-analysis of randomized controlled trials (RCTs) evaluating the efficacy of antibiotic bone cement in reducing prosthetic joint infections in patients undergoing primary total hip or knee arthroplasty.

## Materials and methods

Protocol

This systematic review and meta-analysis was conducted in accordance with the Cochrane Handbook for Systematic Reviews of Interventions. The study is presented as per Preferred Reporting Items for Systematic Reviews and Meta-Analyses (PRISMA) [14]. 

Eligibility criteria

The focus of this systematic review and meta-analysis was on RCTs assessing the efficacy of antibiotic cement in reducing PJI. Inclusion criteria were as follows: 1) RCT, 2) patients undergoing primary or aseptic revision hip or knee arthroplasty, 3) intervention arm of antibiotic cement, 4) comparator of either plain cement or a different number of antibiotics or dose of antibiotics used in the cement, and 5) available in full text in English through the McMaster University library or available for interlibrary loan from an affiliated university. Aseptic revisions were included given that a limited amount of evidence was expected to be available, and thus a broad overview of this intervention was desired. `Exclusion criteria were as follows: 1) studies in which revision surgery was being performed for infection, and 2) overlapping reports from the same study cohort; in this case, the study with the longest follow-up while still being powered for the primary outcome, were included.

Information sources

The search, which was performed on December 19, 2019, included PubMed, MEDLINE, and Embase. All results from database inception to search date were included.

Search

The search strategy included key terms related to arthroplasty, antibiotic cement, infection, and random allocation. Appendix A outlines the full search strategy.

Study selection

After completion of the search, duplicates were removed and the studies imported into Rayyan (Qatar Computing Research Institute, Doha, Qatar). Studies were screened in triplicate by independent reviewers. Studies were screened sequentially in two stages: abstract/title screen and full text. Disagreements at the title and abstract stage were handled with automatic inclusion in the next stage. Discrepancies at the full-text stage were resolved by consensus.

Data collection process

Data were collected using an online collaborative spreadsheet (Google Sheets, Google LLC, Mountain View, California). The spreadsheet was piloted prior to use and adjustments made as necessary. Each reviewer’s data were audited by the other two reviewers for accuracy.

Data items

Data collected included study characteristics, patient demographics, allocation and randomization techniques, and blinding. In addition, details of the intervention and control arms were collected, along with follow-up timelines. Finally, superficial and deep infection, as well as revision outcomes, were collected.

Risk of bias in individual studies

Risk of bias among individual studies was assessed using the Cochrane Risk of Bias Assessment Tool 2.0 [15].

Summary measures

Demographic data is presented using a descriptive statistic, with mean ± standard deviation (SD) for normally distributed data and median ± interquartile range (IQR) for non-parametric data. For comparing dichotomous outcomes, an odds ratio (OR) with 95% Confidence Interval (CI) is presented.

Synthesis of results

A pairwise meta-analysis was performed to compare intervention and control arms for superficial infection, deep infection, and revision rates. A random-effects (RE) model was employed if there was significant heterogeneity, otherwise, a fixed-effects (FE) model was utilized. Heterogeneity was assessed using the chi-squared statistic, presented as an I2 value, and considered to be significant if p < 0.1. For all other tests, significance was set at the p < 0.05 level a priori.

## Results

Study selection

**Figure 1:**
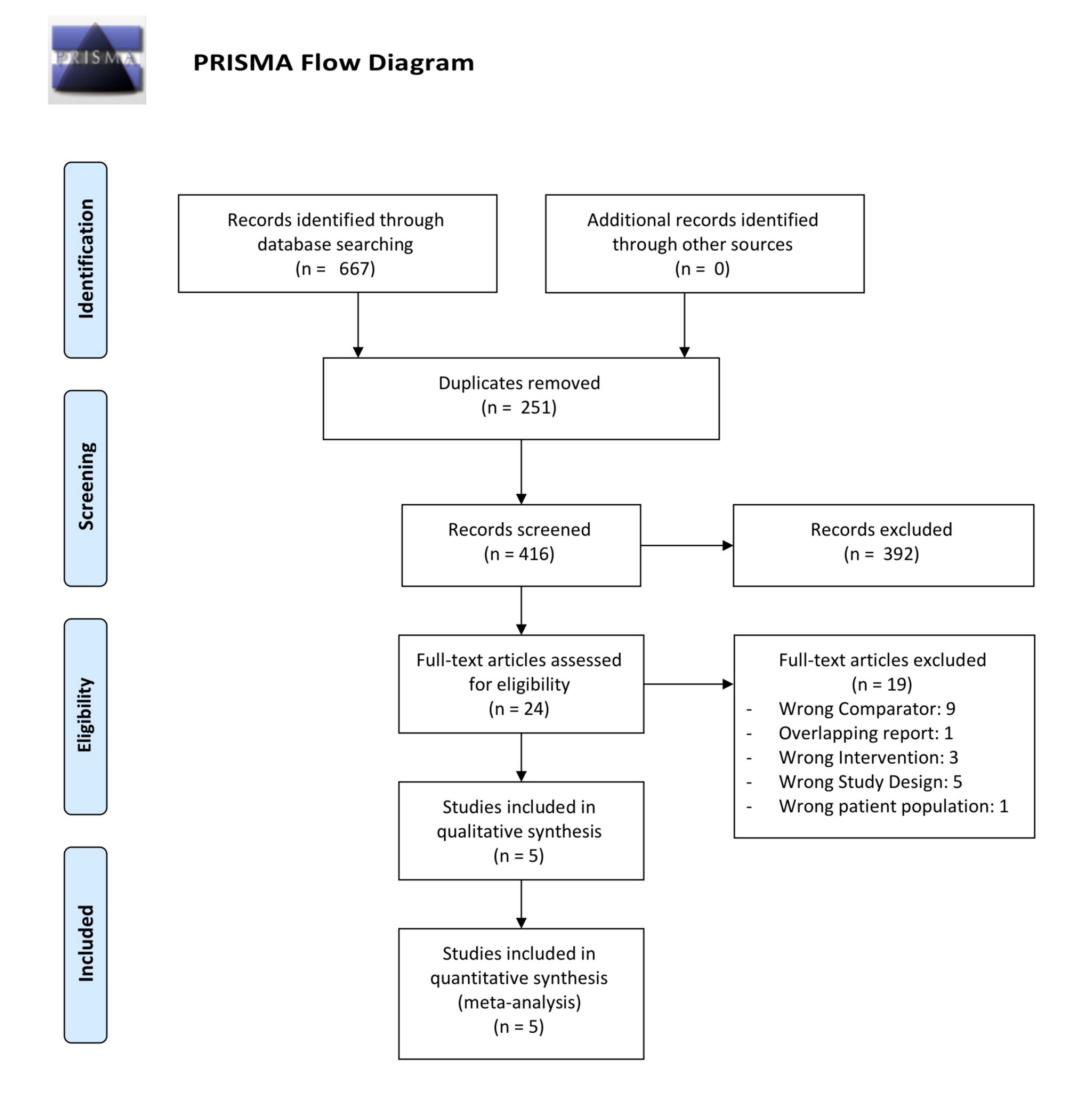
PRISMA flow diagram

The search strategy returned 667 results. Ultimately, five RCTs were included. See Figure 1 for the PRISMA flow diagram [14].

Study characteristics

The five included studies were published between 2001 and 2016 and enrolled a total of 4,397 patients [16-20]. Four studies compared antibiotic cement to plain cement while one study compared high-dose dual-antibiotic (HDDA) cement to low-dose single-antibiotic (LDSA) cement [16-20]. Overall, 2,643 patients underwent procedures with antibiotic cement while 1,754 underwent procedures with plain cement. Seventy percent of enrolled patients were female (3,076/4,397), and the weighted mean age of all included patients was 76.4 years old (range: 68-82.96). Three studies assessed patients undergoing primary TKA (n = 3549), one study assessed those undergoing aseptic revision TKA (n = 183), and one study assessed patients undergoing hip hemiarthroplasty for fracture (n = 848). Cefuroxime was the active antibiotic in one study while vancomycin, gentamicin, erythromycin were used in one study each. One study used a combination of gentamicin and clindamycin in the HDDA group. Table 1 details the characteristics of the included studies.

**Table 1:** Characteristics of included studies

Lead Author	Year	Country	Sample Size	Female (%)	Mean Age (years)
Chiu [18]	2001	China	78	32.1	70.6
Chiu [20]	2002	China	340	30.3	69.0
Chiu [19]	2009	China	183	36.6	70.5
Hinarejos [17]	2013	Spain	2948	76.3	75.9
Sprowson [16]	2016	United Kingdom	848	74.5	82.6

Synthesis of results

Superficial Infection

**Figure 2:**
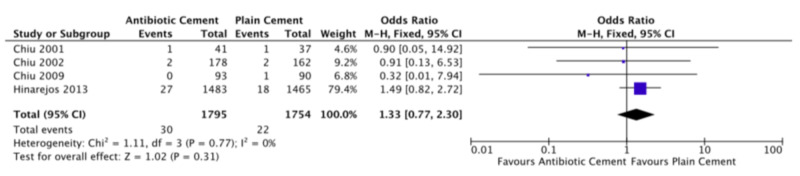
Forest plot of superficial infection rates

The four studies comparing antibiotic cement to plain cement all reported superficial infection rates [17-20]. None of the studies found a significant effect for antibiotic cement. The pooled superficial infection rate was 1.7% (30/1795) in the antibiotic cement group as compared to 1.3% (22/1754) in the plain cement group. The pooled estimate, comparing antibiotic cement to plain cement, revealed an OR of 1.33 (FE, 95% CI: 0.77-2.30, p = 0.3), slightly favoring plain cement though this was not significant. There was no heterogeneity (I2 = 0%). Figure 2 displays the forest plot for superficial infection. The study comparing HDDA to LDSA cement found a superficial infection rate of 0.6% (2/360) in the HDDA group as compared to 1.9% (7/376) in the LDSA group (OR: 0.29, 95% CI: 0.06-1.43, p = 0.13) [16].

Deep Infection

**Figure 3:**
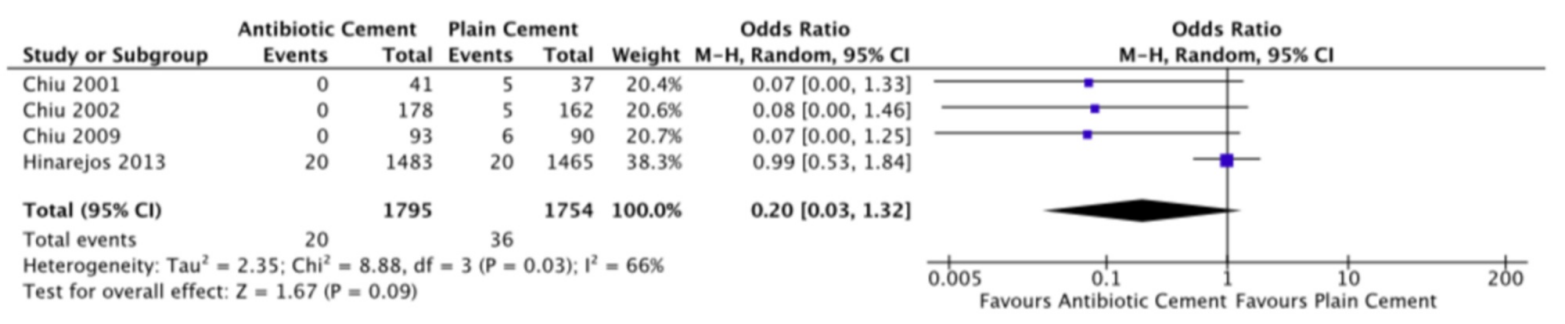
Forest plot of deep infection rates

All studies reported deep infection rates. Among the studies comparing antibiotic cement to plain cement, none found a significant effect, though all trended in favor of antibiotic cement [17-20]. Pooled deep infection rate for antibiotic cement was 1.1% (20/1795) as compared to 2.1% (36/1754) for plain cement (OR: 0.20, RE, 95%CI: 0.03-1.32, p = 0.09). There was moderate heterogeneity (I2 = 66%). Figure 3 displays the forest plot for deep infection. The study comparing HDDA to LDSA cement found a significantly lower rate of infection with HDDA (1.1% vs. 3.5%, OR: 0.31, 95% CI: 0.09-0.88, p = 0.041) [16].

Risk of Bias Among Included Studies

Of the five included studies, four were found to be at an overall high risk of bias while one study was rated “some concerns” [16-20]. Blinding and random sequence generation were the most domains most frequently at a high risk of bias while all studies had a low risk of bias for missing outcome data. Table 2 details the risk of bias assessment.

**Table 2:** Risk of bias across included studies

Lead Author	Randomization Process	Blinding	Intention-to-treat Analysis	Missing Outcome Data	Measurement of Outcome	Selection of Reported Result	Overall Risk of Bias
Chiu 2001 [18]	High risk	High risk	Some concerns	Low risk	Some concerns	Some concerns	High risk
Chiu 2002 [20]	High risk	High risk	Some concerns	Low risk	Some concerns	Some concerns	High risk
Chiu 2009 [19]	High risk	High risk	Some concerns	Low risk	Some concerns	Some concerns	High risk
Hinarejos 2013 [17]	Low risk	Some concerns	High risk	Low risk	Some concerns	Low risk	High risk
Sprowson 2016 [16]	Some concerns	Low risk	Low risk	Low risk	Low risk	Low risk	Some concerns

Additional Analyses

Given that one of the studies included in the quantitative meta-analysis included patients undergoing revision surgery, we performed a post-hoc sensitivity analysis by repeating the meta-analysis without including that study [19]. The results were very similar to the original analysis though with less precision (OR: 0.26, 95%CI: 0.03 to 2.17, p = 0.21). See Appendix B for the forest plot for this sensitivity analysis.

## Discussion

The findings of this systematic review and meta-analysis reveal that despite a total of 4,397 patients having been randomized to receive antibiotic cement or plain cement, the overall effect of antibiotic cement remains unclear. While the pooled effect estimate suggests a very large reduction in rates of PJI, this point estimate is associated with massive confidence intervals that cross the line of no effect. Thus, further high-quality evidence is needed in the form of very large, adequately powered, multicenter, and pragmatic RCTs that seek to answer this question definitively.

The presence of deep infection can be caused by either direct infiltration from skin flora and contaminants or hematogenous spread from other sources. In the setting of acute periprosthetic infection, it is assumed that nearly all infections are caused through direct infiltration secondary to the formation of a biofilm [21]. The biofilm reliably forms through four steps: formation of a conditional film, microbial mass transport, anchoring through exopolymer production, and eventual growth of adhering micro-organisms [22]. In-vitro models have demonstrated that antibiotic cement reduces biofilm formation on cement discs after 24 hours [23]. This has been theorized to have occurred secondary to the inhibition of microbial mass transport, as the formation of a conditional film is unaffected by the presence of antibiotic bone cement. Moreover, although the diffusion properties of different antibiotic cement mixtures vary, the general patterns are the same: a large amount of antibiotic is released in the first 24-48 hours, followed by slow diffusion over the next days to weeks postoperatively [22].

Given that antibiotic cement primarily enacts its effects over the course of many weeks, it is logical that it would not prevent infection by inhibiting the creation of the conditional biofilm but rather by reducing bacterial transport to eventual biofilm formation. This is consistent with our finding that antibiotic cement does not reduce the risk of superficial infections, with an infection rate of 1.7% and 1.3% in the antibiotic and plain cement groups, respectively. Existing bacteria that colonize the skin during the perioperative period are responsible for the majority of superficial infections [24]. Therefore, antibiotic cement, which works over a period of days to a week, would not likely prevent contamination of the wound with these surface organisms. Rather, perioperative intravenous antibiotic prophylaxis and diligent sterile technique are more likely to reduce superficial surgical site infections [21,25-26].

The dose and type of antibiotic may be an important consideration when selecting an appropriate cement. The studies included in this review utilized five different antibiotics at varying doses, some of which are no longer in common use, which may have contributed to the levels of heterogeneity and imprecision. Furthermore, the rates of elution even among the same antibiotics is different in cement from different manufacturers. Previous literature has discussed the importance of fast-setting cement in the context of arthroplasty [27]. Furthermore, the single study that compared two different doses of antibiotic cement found that the high-dose, dual antibiotic cement was associated with a significantly lower rate of infection as compared to a low-dose, single antibiotic cement [16]. Generally, less than 1 g of antibiotics per 40 g of cement is considered to be a low dose.

Given the sheer volume of total joint replacements, the findings of this study need to be contextualized within a resource-limited health care system. Costs for antibiotic cement have been reported at nearly $300 more per case as compared to plain cement ($416 vs $117) while the total 90-day costs for a patient requiring revision surgery secondary to PJI are approximately USD 30,000 higher than one without PJI [28-29]. Currently, the annual cost of revision surgeries in Canada is estimated at 163 million Canadian dollars (CAD), and infection is the leading cause of revision surgery, accounting for 31.8% of all revisions [1]. Thus, even a modest relative risk reduction of 25% would result in annual savings of nearly 15 million CAD in Canada alone.

The strengths of our review lie in our rigorous methodological approach and the high quality of evidence included. Our review only included the highest quality evidence in randomized controlled trials, we performed title, abstract, and full-text screening in triplicate, and we performed random spot checks on abstraction to ensure accuracy.

This study is limited by the heterogeneity of various interventions such as primary, revision, and hemiarthroplasty. However, all studies showed a relative reduction in deep PJI regardless of the antibiotic used. Furthermore, though the included studies were all RCTs, the number of studies is limited and the pooled estimate is associated with high imprecision.

## Conclusions

Overall, pooled data on the best available evidence demonstrates a high level of imprecision. Thus, there is a need for large, pragmatic trials on this topic in order to provide a definitive answer to this important question. Future trials should consider specific questions, such as the effective antibiotic dose to be used, as well as cost-utility analyses.

## Figures and Tables

**Figure 4:**
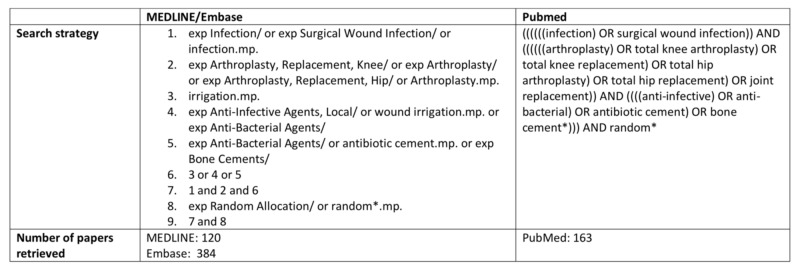
Search strategy

**Figure 5:**
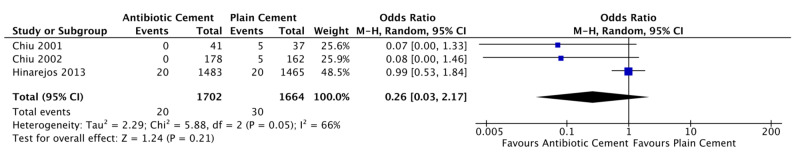
Forest plot of the post-hoc sensitivity analysis

## Appendices

Appendix A 

Appendix B
